# Supplementary material for: Gold nanocarriers for transport of oligonucleotides across brain endothelial cells
Source: PLoS One. 2020 Sep 17;15(9):e0236611. doi: 10.1371/journal.pone.0236611 (PMC7498062; doi:10.1371/journal.pone.0236611)
Supplement: S1 File — (DOCX) [file pone.0236611.s001.docx]

**Supplementary Figure-1**

Au

-S-S-C6–AATATCGCGGACAGAAGACGGAAACCAAGGCAGAGCTTTT 3’

TTATAGCGCCTGTCTTCTGCCTTTGGTTCCGTCTCGAAAA 5’

Diagram of the 40nt ds oligonucleotide attached to the gold NPs. The initial exchange reaction attached a thiolated 20nt ssDNA (orange) to the gold core. This was subsequently hybridised with a 40nt ssDNA (black) to extend the attached oligonucleotide which was then filled with a 20nt ssDNA oligonucleotide (blue) to produce the 40nt dsDNA attachment.

**Supplementary Figure-2**


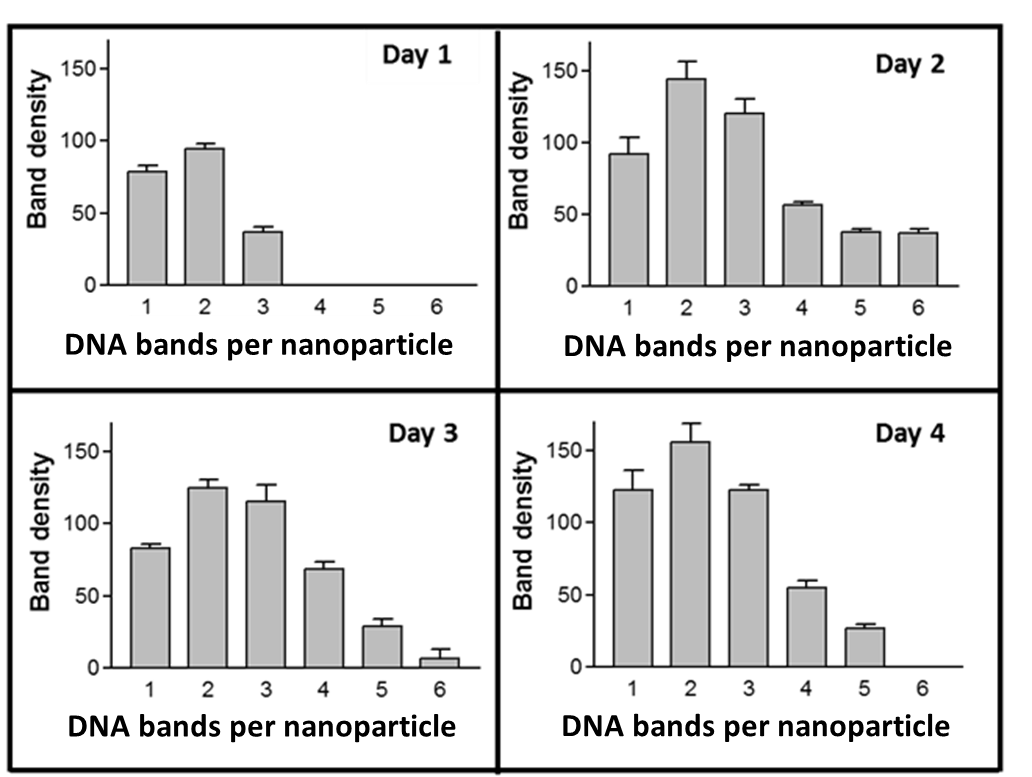


Analysis of band density from the 4 days-place exchange reaction using ImageJ. The highest quantity of NP-DNA bands were seen at day-2 of the reaction.

**Supplementary Figure-3**

**
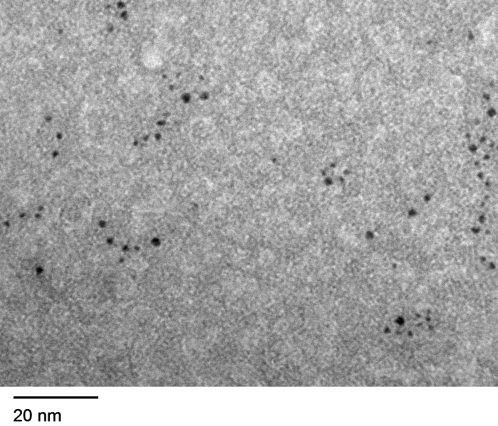
**

**A**

**B**

| **Oligonucleotides bound to the NPs** | **Diameter of gold core of NPs (nm)** | **Hydrodynamic diameter (nm)** |
| --- | --- | --- |
| **0** | **1.29 ± 0.3** | **1.8** |
| **1** | **1.17 ± 0.26** | **3.4** |
| **2** | **1.36 ± 0.42** | **4.4** |
| **3** | **1.36 ± 0.38** | **4.9** |
| **4** | **1.29 ± 0.33** | **6.2** |

Core size and hydrodynamic diameter of NP-DNA with different stoichiometries. (A) Transmission electron micrograph of the Gal-NPs with 1 DNA oligonucleotide attached showing the gold core of the nanoparticles. (B). A table of the sizes of the gold cores of NPs with different numbers of bound 20nt ssDNA oligonucleotides (mean ± sd) derived from direct measurement of TEM images of at least 200 NPs each. An estimate of the hydrodynamic diameters was derived from elution volumes on FPLC – see Supplementary Fig. S4 and Fig. 1B.

**Supplementary Figure-4**


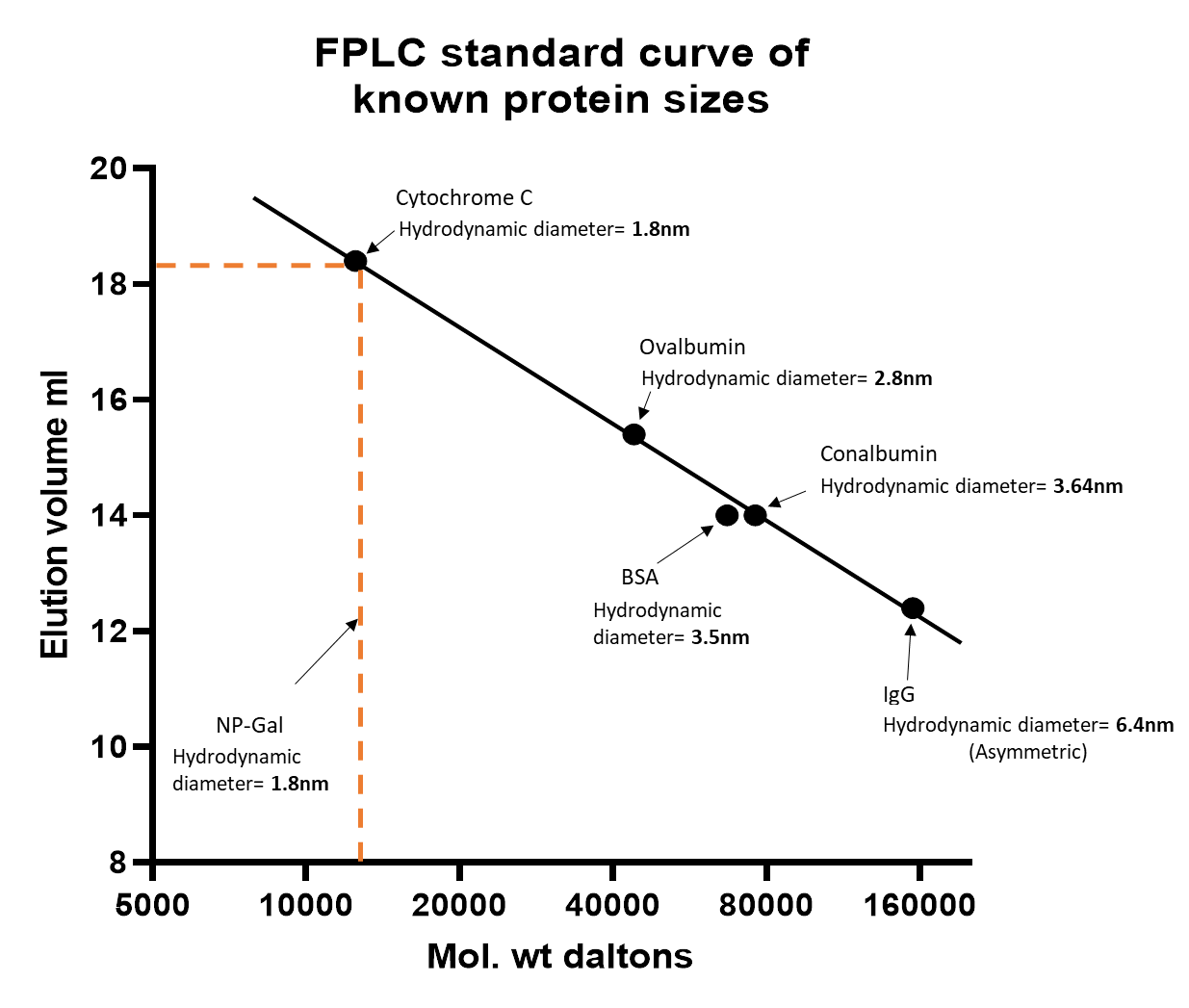


Calibration of the Superdex 200 10/300 GL column, using proteins of known hydrodynamic diameter. The effective diameters of NPs were derived from the standard curve, as illustrated for the base nanoparticle NP-Gal.

**Supplementary Figure-5**


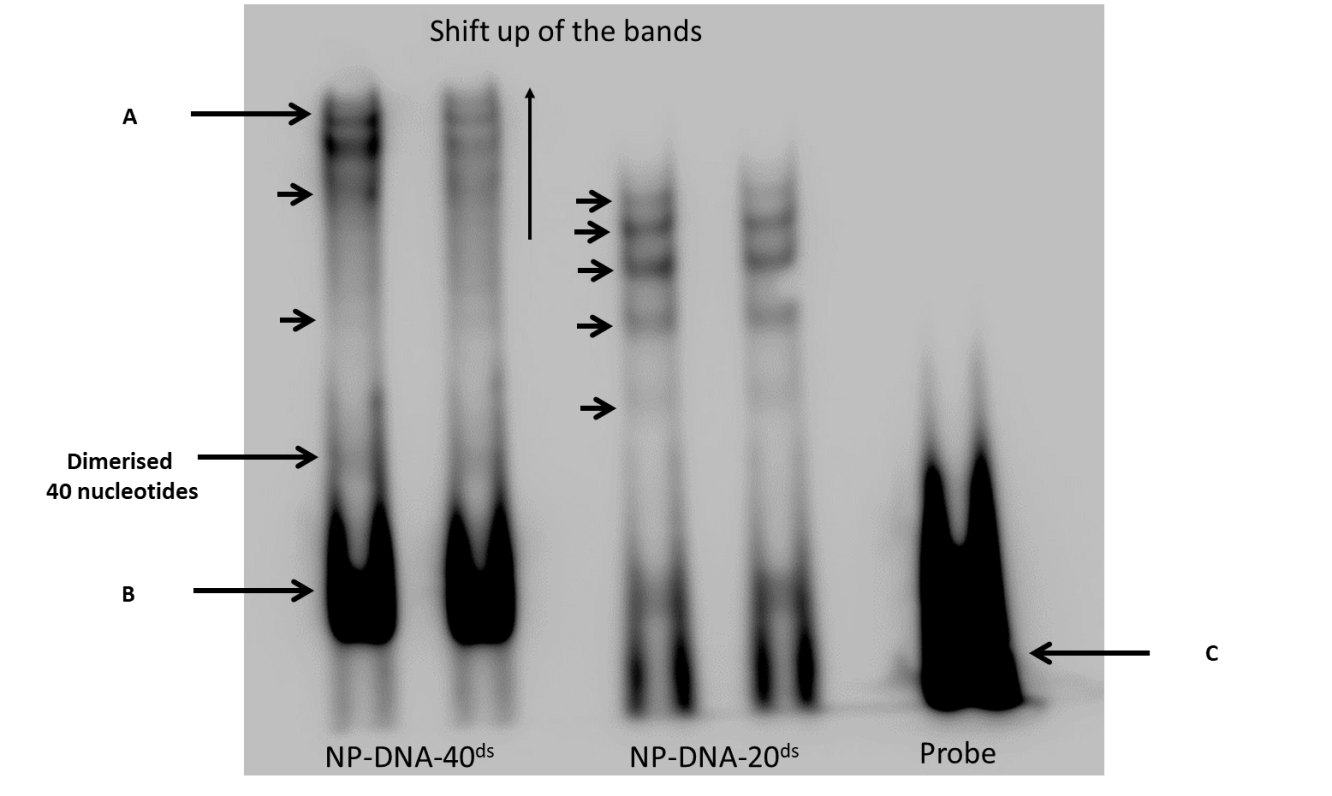


EMSA of NP-DNA-40^ds^ compared with NP-DNA20^ds^. The 20nt extension of the attached oligonucleotides causes the NP-DNA conjugates to increase in size and the bands are shifted up the gel (A). The pairs of bands are duplicates with double the amount of sample in the left hand lanes. Free biotinylated probe (C) and probe bound to free oligonucleotides(B) are located at the bottom of the 5% gel.

**Supplementary Figure-6**

hCMEC/D3 cell viability measured by Alamar blue assay when exposed to 8, 16, 32 and 50 µg/mL of NP-DNA-40^LO^, NP-DNA-40^HI^ and NP-Gal for 24 hours (n=3). Positive control of cell death is 30 µg/ml digitonin (30 min treatment). Notes: Data are shown as ratio of E570 (reduced form) /E620 (oxidised form) which represents the maximum cell viability. Tukey's multiple comparisons test showed significant difference for NP-Gal and NP-DNA-40^LO^ applied at 50 µg/mL (p-value: * 0.026, **0.0084.

**Supplementary Figure-7**

*Number of nanoparticles observed in each astrocyte for NP-DNA-40^LO^ and NP-DNA-40^HI^ compared with NP-Gal. Three experiments were performed, each individual experiment having three technical repeats. Tukey's multiple comparisons test showed significant difference for NP-Gal compared to NP-DNA-40^LO^ and NP-DNA-40^HI^ (**** P <0.0001).*
