# Supplementary material for: Gold nanocarriers for transport of oligonucleotides across brain endothelial cells
Source: PLoS One. 2020 Sep 17;15(9):e0236611. doi: 10.1371/journal.pone.0236611 (PMC7498062; doi:10.1371/journal.pone.0236611)
Supplement: S1 Raw images — (PDF) [file pone.0236611.s002.pdf]

Original blot image for Fig. 1A, (Geldoc)

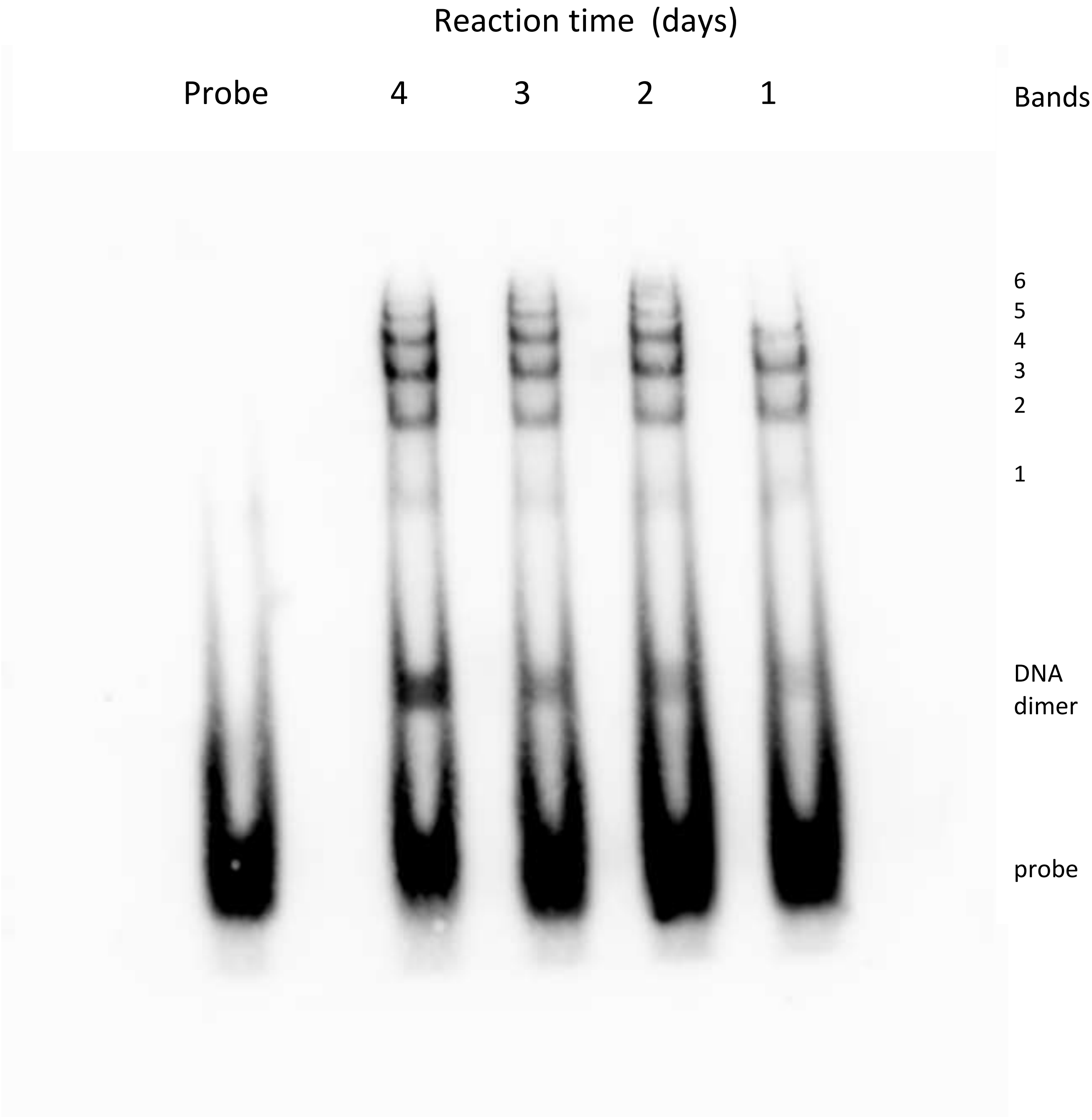

Blot of EMSA of exchange reaction probed with a biotinylated oligonucleotide complementary to the ssDNA oligonucleotide attached to the NPs.

Each lane is the reaction products of an exchange reaction carried out for the number of days stated. The bands show gold nanoparticles with 1-6 covalently-bound oligonucleotides.. DNA dimer is the oxidised (unreacted) oligonucleotide.

Original blot image for Fig. 1C, (Geldoc)

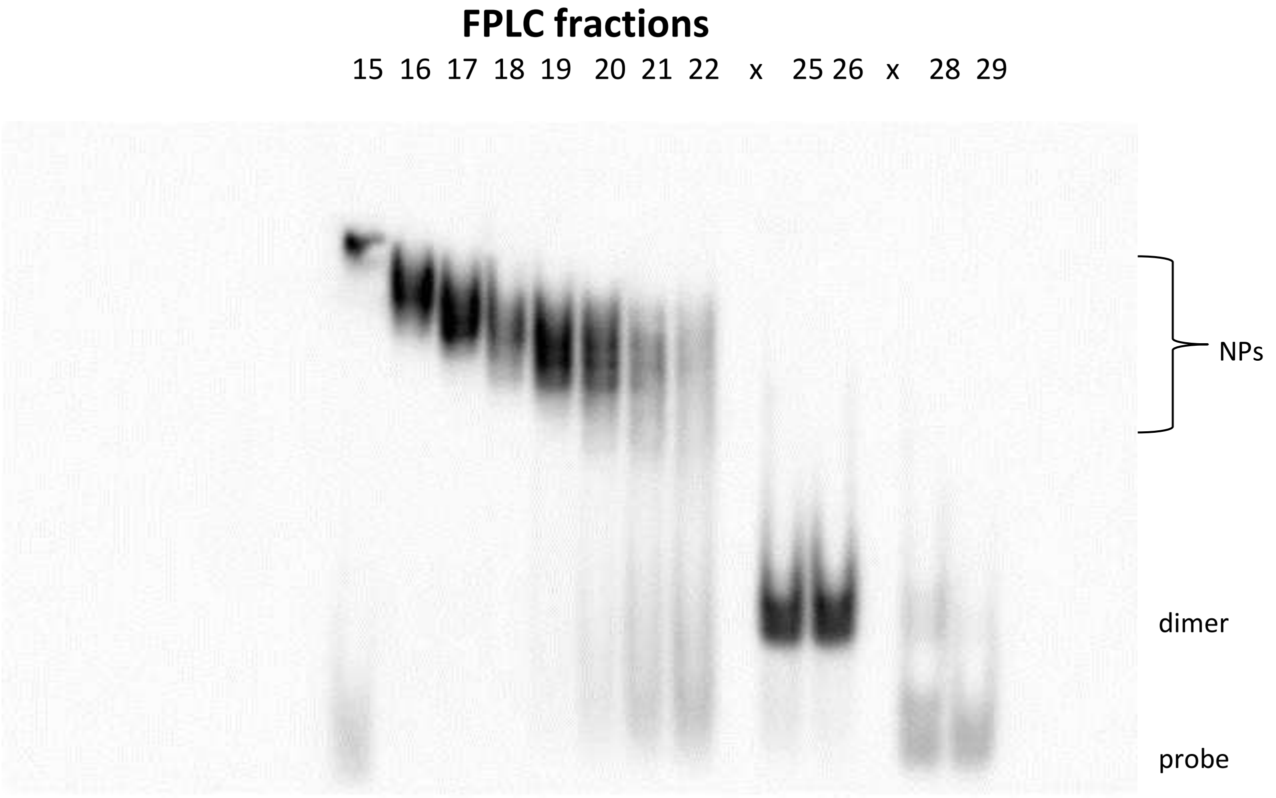

Blot of EMSA of FPLC fractions (15-22, 25&26, 28&29) corresponding to the fraction numbers shown in Fig. 1B. Blots were probed with a biotinylated oligonucleotide complementary to the oligonucleotide attached to the nanoparticles (NPs) . Unreacted DNA dimer is in fractions 25 and 26. There is excess free probe, in fractions with lower amounts of oligonucleotide.

Original blot image for supplementary Fig. S5. (Geldoc)

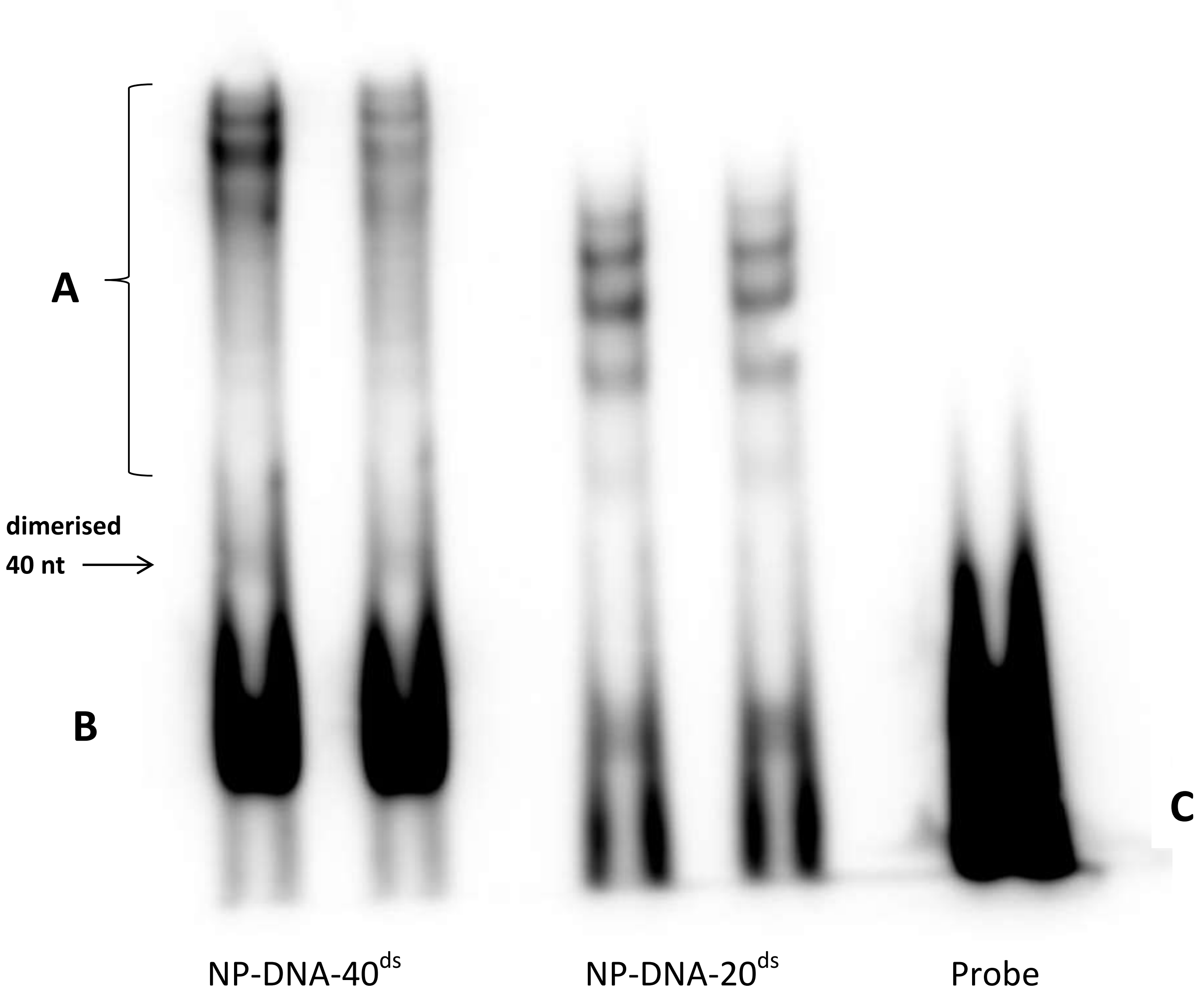

S5 Fig. EMSA of NP-DNA-40^{ds} compared with NP-DNA20^{ds}. The 20nt extension of the attached oligonucleotides causes the NP-DNA conjugates to increase in size and the bands are shifted up the gel (A). The pairs of lanes are duplicates with double the amount of sample in the left hand lanes. Free biotinylated probe (C) and probe bound to free oligonucleotides (B) are located at the bottom of the 5% gel.
