# Supplementary material for: Gold nanocarriers for transport of oligonucleotides across brain endothelial cells
Source: PLoS One. 2020 Sep 17;15(9):e0236611. doi: 10.1371/journal.pone.0236611 (PMC7498062; doi:10.1371/journal.pone.0236611)
Supplement: Attachment — Submitted filename: Rebuttal letter.doc [file pone.0236611.s003.doc]

**Gold nanocarriers for transport of oligonucleotides across brain endothelial cells. (revised)**

Faculty of Science Technology Engineering and Maths

Life Sciences

The Open University
Walton Hall
Milton Keynes
MK7 6AA
United Kingdom

Tel +44 (0) 1908 659226
email: [David.Male@open.ac.uk](mailto:David.Male@open.ac.uk)
[www.open.ac.uk](http://www.open.ac.uk/)

Professor David Male Immunology and Cell Biology

Dr Yi Ciao,

Academic editor,

Plos ONE

20^th^ August 2020

We thank the editor and reviewers for their careful consideration of this paper. In response we have added an additional supplementary figure and table, made substantial changes to the introduction, results and discussion and added 4 additional references, as detailed below:

Response to the editorial comment on additional cytotoxicity assays:

*This class of NPs with a covalently bound C2- sugar coat and cargo has been approved by the FDA for delivery to humans and phase 1 and 2 clinical trials carried out with an insulin cargo. The FDA submission produced by Midatech Pharma plc included cytotoxicity and pathology studies in rats, mini-pigs, and beagles and genotoxicity in S. typhimurium. For example.*

*Single dose oral treatment of Wistar rats (12mg/Kg oral) in Wistar rats – ‘No pathological changes were detected in brain, heart, lungs, or spleen’:*

*Repeated oral dose toxicity of Wistar rats, up to 1.5mg/Kg over 14 days and followed over 29 days – ‘there were no treatment-related changes in hematology, clinical chemistry, gross pathology or histopathology’.*

*In genotoxicity tests (micronucleus and comet assay), it has been shown that 5nm gold NPs up to 250μg/ml showed no effect in either assay, provided they were neutral or negatively charged (new ref. 36). We had previously carried out NP cytotoxicity studies on hCMEC/D3 cells with an MTT assay (ref.12)and shown no cytotoxicity at the doses used here.*

*Additions have been made to the introduction and discussion, to highlight that other assays in vitro and in vivo have been performed with these NPs, albeit with a different cargo*

Response to reviewers.

Reviewer #1

1. The mechanisms of the gold nanocarriers transport of oligonucleotides should be further enhanced.

*We have previously investigated the mechanisms of gold nanocarrier transport across hCMEC/D3 cells (ref. 23). The section in the discussion, which cited this study has been expanded and correlated with the new results in this paper.*

2. The SEM images of the gold nanoparticle should be added.

*We think the reviewer means transmission EM images, since these nanoparticles are too small to resolve in SEM. An example TEM image of the NPs has now been added as* ***supplementary figure 3*** *together with a table of core diameters and hydrodynamic diameters for NPs with 1-4 20nt ssDNA oligonucleotides attached. The core size does not change significantly, which implies no aggregation of the NPs when DNA is attached. The hydrodynamic diameter of the NPs is more accurately determined by FPLC as already described in the manuscript and supplementary fig. 3.*

3. Since brain cells were very sensitive, can the author further discuss the advantages of this technology.

*Additional discussion and references have been added, which also address the points on safety also raised by the editor.*

Reviewer #2 … But there is a safety concern, what about the binding affinity between the AuNPs and oligonucleotides? Will there be a side effect due to the leakage during the transport process?

*Additional notes have been added in the discussion, on the stability of the nanoparticles in the conditions normally found in the cytosol or vesicles. The data indicating that relatively little DNA is detected in the endothelial cells (Fig 6) has been highlighted and discussed.The point is made that oligonucleotides detected in the endothelial cells may still be in transit,(attached to the NPs) and not free DNA.*

We hope that this additional data and discussion answers the points raised by the reviewers, and look forward to hearing from you.

Yours sincerely,
